# Supplementary figures and images for: Control of mRNA translation by dynamic ribosome modification
Source: PLoS Genet. 2020 Jun 25;16(6):e1008837. doi: 10.1371/journal.pgen.1008837 (PMC7343187; doi:10.1371/journal.pgen.1008837)

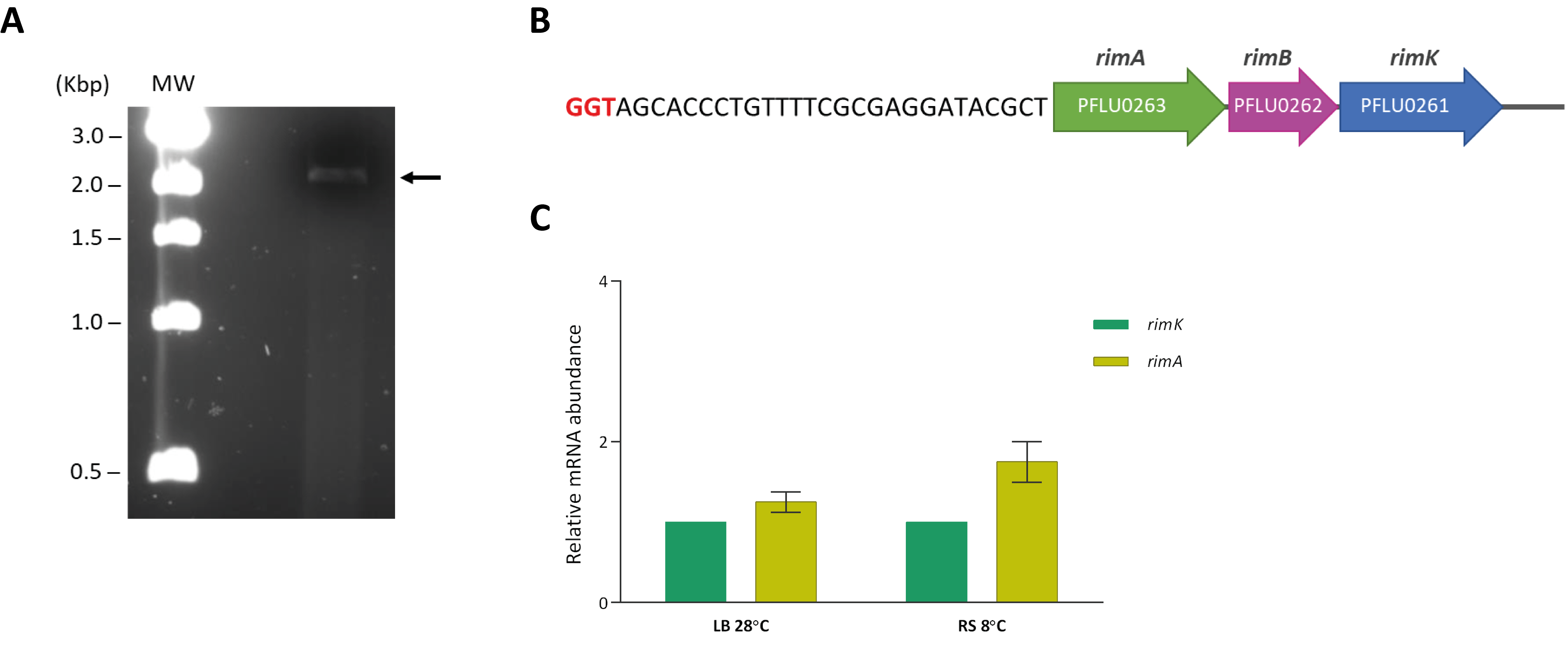

Supplement: S1 Fig — (A) PCR of the rim operon from cDNA. The indicated band shows the position of the 2.2Kbp PCR product resulting from the amplification of the rimABK operon. (B) Determining the transcription start site of the rimABK operon. A cartoon representation of the rimABK operon and upstream region. Transcription initiation begins at one of the three nucleotides highlighted in red. The 5’ RACE methodology does not allow discrimination between these nucleotides. (C) rimA mRNA relative abundance does not substantially differ from that of rimK. mRNA abundance (qRT PCR data) relative to cells grown overnight in LB and transferred to LB at 28°C and RS at 8°C for 45 minutes prior to sampling. RS–carbon free ‘Rooting Solution’ [19]. Data are presented +/- the standard error of three replicates. The experiment was repeated three times independently and a representative is shown. (TIF) [file pgen.1008837.s001.tif]

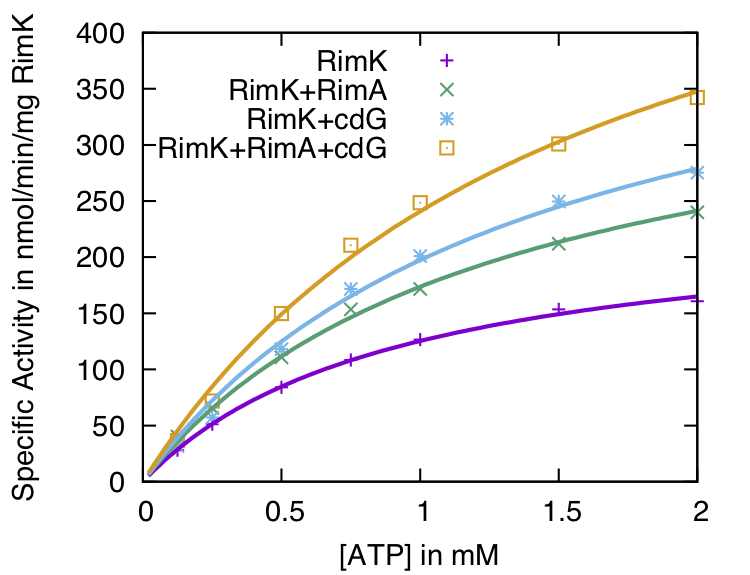

Supplement: S2 Fig — This plot shows the experimental data from Fig 2E as points and the curves are computed from kinetic models based on RimA and cdG binding to RimK (S1 Table). Under the assumption that RimK can exist in two states, RimK and RimK*, with different activities and that RimK* achieved either by binding cdG or RimA, we simultaneously optimised all the parameters in the system (percentage of RimK*, kcat* and Km*). This plot shows that a simple two state model is consistent with the data. However, under this hypothesis and with [RimK] = 1 μM, [RimA] = 1 μM, [cdG] = 25 μM, the best fit to the ATPase data is achieved for an equilibrium dissociation constant (Kd) of 83.2 nM between RimK and RimA and 15.1 μM between RimK and cdG. This it at odds with independent measurements that estimate Kd between RimK and cdG to be about 1 μM [19]. This suggests that the simple two state system is unlikely and that RimK can exist in at least four different activation states (RimK, RimK.RimA, RimK.cdG and RimK.cdG.RimA, S1 Table). This model of RimK ATPase activity results in an insignificantly small better fit to the ATPase activity data but with a Kd between RimK and cdG of 1 μM and a Kd between RimK and RimA of 0.2 μM. (TIFF) [file pgen.1008837.s002.tiff]

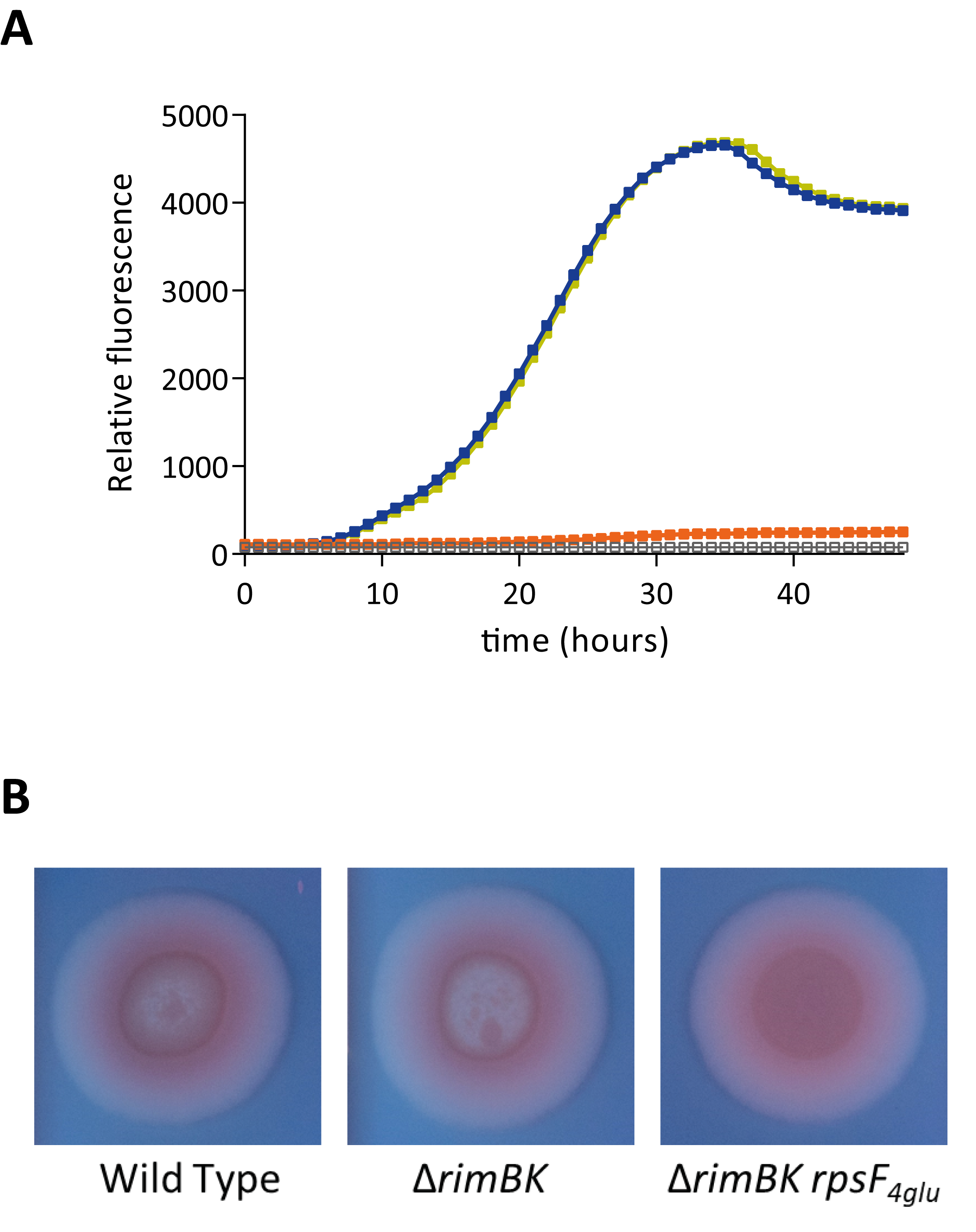

Supplement: S3 Fig — (A) Relative fluorescence (arbitrary units) at A460 of SBW25 mutant strains. WT SBW25 is shown in gold, ΔrimBK in blue and ΔrimBK rpsF4glu in orange. (B) Colony morphology resulting from 72-hour growth of 5 μL spots of the indicated strains, on M9-pyruvate plates containing 0.004% Congo Red dye and 100 μM FeCl2. (TIF) [file pgen.1008837.s003.tif]

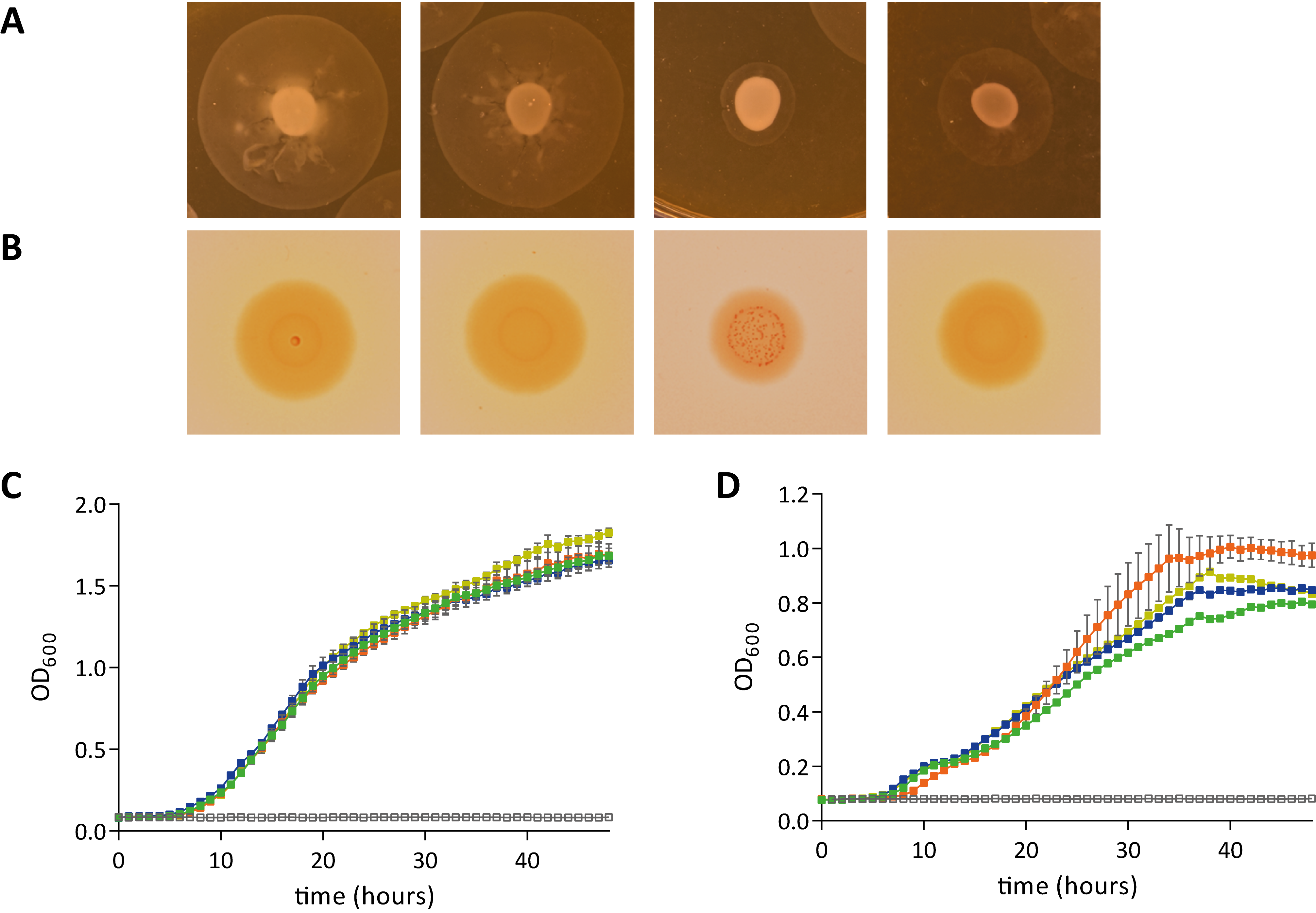

Supplement: S4 Fig — (A) Glutamation of RpsF at the C-terminus influences swarming motility. Overnight growth on swarm diameter plates containing 0.3% w/v agar and 0.05% Congo Red dye. (B) Colony morphology resulting from overnight growth of 5 μL spots of the indicated strains, on KB agar plates containing 0.004% Congo Red dye. The presence of four C-terminal glutamates on RpsF results in smaller colonies and increased dye binding. (C) Growth curves in KB medium and (D) in M9-pyruvate medium. In both charts, WT SBW25 is shown in gold, ΔrimBK in blue, ΔrimBK rpsF4glu in orange and ΔrimBK rpsF10glu in green. The black line shows the absorbance of the uninoculated media. (TIF) [file pgen.1008837.s004.tif]

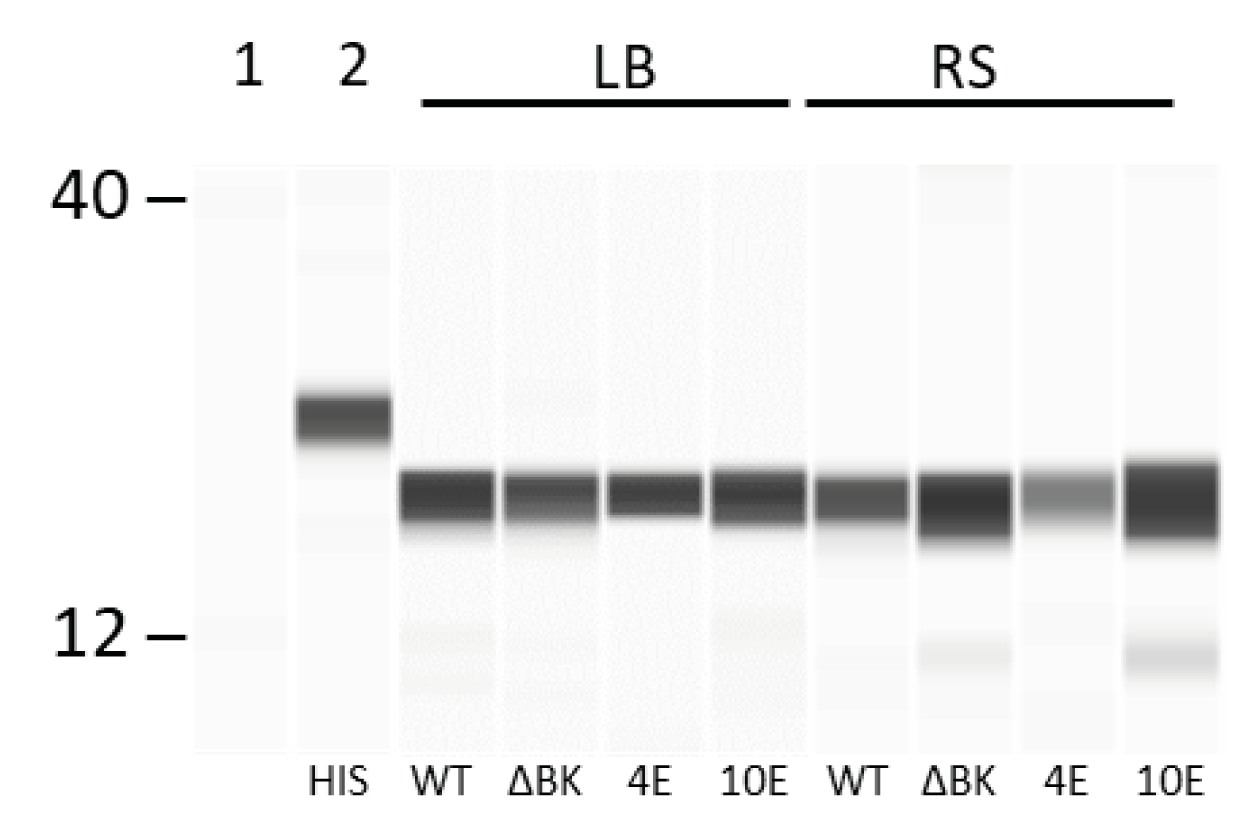

Supplement: S5 Fig — Anti-RpsF immunoblot of ribosomes from different genetic backgrounds. Lane 1, molecular weight marker track (markers not resolved at this resolution). Lane 2, Purified hexa-histidine RpsF control. Cells were grown in either Lysogenic Broth media (denoted LB) at 28°C or Rooting Solution (denoted RS; [19]) at 4°C. WT, ΔBK, 4E and 10E denote ribosomes purified from wild-type, ΔrimBK, ΔrimBK rpsF4glu and ΔrimBK rpsF10glu respectively. (TIF) [file pgen.1008837.s005.tif]

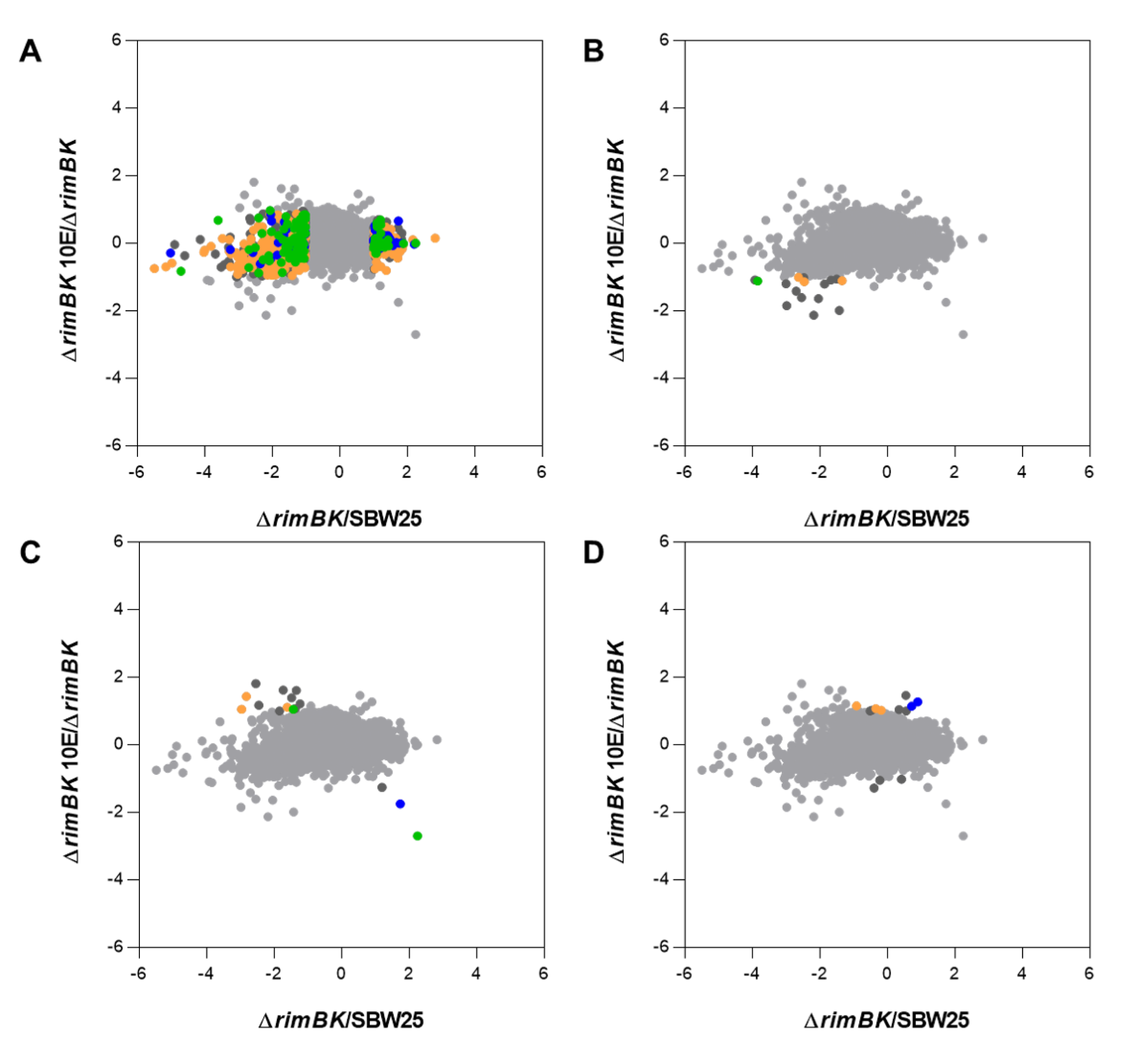

Supplement: S7 Fig — (A-C) Highlighted regions containing significantly (>1.0 log2) affected class 1, 2 and 3 genes respectively. (D) Genes significantly (>1.0 log2) affected by rpsF glutamation but not significantly affected by rimBK deletion. Highlighted genes are listed in S4 Table and colour-coded according to their COG classifications: yellow = metabolism; green = cellular processes and signalling; blue = information storage and processing; dark grey = poorly characterised. (TIF) [file pgen.1008837.s007.tif]

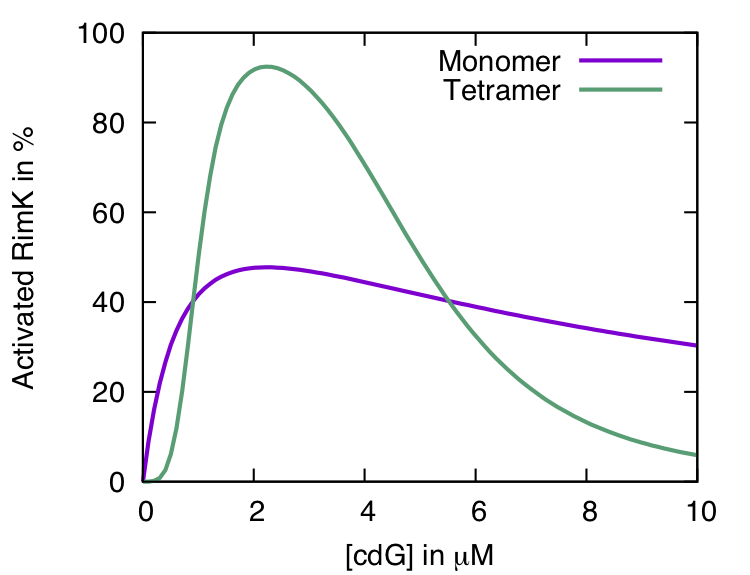

Supplement: S8 Fig — Our experimental data show that both cdG and RimA increase the ATPase and glutamate activity of RimK in the absence of RimB. A model of RimA and cdG binding to RimK, leading to a state of higher activity, is consistent with this data (S2 Fig). In the presence of RimB, however, RimA becomes catalytically active, binding to and modifying cdG. Given the experimental data, a reasonable hypothesis is that this RimA activity promotes, either directly or indirectly, the dissociation from RimK. Here we model the effect of stimulating the RimK.RimA complex by cdG binding to RimK (Kd = 1 μM) and its inhibition by binding cdG to RimA (Kd = 5 μM) for different assumptions of different complex arrangements for RimK.RimA. (TIFF) [file pgen.1008837.s008.tiff]
