# Supplementary material for: Control of mRNA translation by dynamic ribosome modification
Source: PLoS Genet. 2020 Jun 25;16(6):e1008837. doi: 10.1371/journal.pgen.1008837 (PMC7343187; doi:10.1371/journal.pgen.1008837)
Supplement: S1 Table — (DOCX) [file pgen.1008837.s009.docx]

**S1 Table Kinetic models for RimK ATPase activity**

| **Model** | **Chemical reactions** | **Constraints** |
| --- | --- | --- |
| **Two state RimK** | $RimK+RimA \rightleftarrows{RimK}^{*}$  $RimK+cdG \rightleftarrows{RimK}^{*}$  $RimK+ATP \rightleftarrows{RimK.ATP} \to RimK+ADP+P_{i}$  ${RimK}^{*}+ATP \rightleftarrows{{RimK}^{*}.ATP} \to{RimK}^{*}+ADP+P_{i}$ | $RimK+{RimK}^{*}={RimK}^{Total}$ |
| **Three state RimK** | $RimK+RimA \rightleftarrows{RimK}^{1}$  $RimK+cdG \rightleftarrows{RimK}^{2}$  ${RimK}^{1}+cdG \rightleftarrows{RimK}^{2}$  ${RimK}^{2}+RimA \rightleftarrows{RimK}^{2}$  $RimK+ATP \rightleftarrows{RimK.ATP} \to RimK+ADP+P_{i}$  ${RimK}^{1}+ATP \rightleftarrows{{RimK}^{1}.ATP} \to{RimK}^{1}+ADP+P_{i}$  ${RimK}^{2}+ATP \rightleftarrows{{RimK}^{2}.ATP} \to{RimK}^{2}+ADP+P_{i}$ | $RimK+{RimK}^{1}+ {RimK}^{2}={RimK}^{Total}$  $K_{d:RimK.RimA}K_{d:{RimK}^{1}.cdG}=K_{d:RimK.cdG}K_{d:{RimK}^{2}.RimA}$ |
| **Four state RimK** | $RimK+RimA \rightleftarrows{RimK}^{1}$  $RimK+cdG \rightleftarrows{RimK}^{2}$  ${RimK}^{1}+cdG \rightleftarrows{RimK}^{3}$  ${RimK}^{2}+RimA \rightleftarrows{RimK}^{3}$  $RimK+ATP \rightleftarrows{RimK.ATP} \to RimK+ADP+P_{i}$  ${RimK}^{1}+ATP \rightleftarrows{{RimK}^{1}.ATP} \to{RimK}^{1}+ADP+P_{i}$  ${RimK}^{2}+ATP \rightleftarrows{{RimK}^{2}.ATP} \to{RimK}^{2}+ADP+P_{i}$  ${RimK}^{3}+ATP \rightleftarrows{{RimK}^{3}.ATP} \to{RimK}^{3}+ADP+P_{i}$ | $RimK+{RimK}^{1}+ {RimK}^{2}+ {RimK}^{3}={RimK}^{Total}$  $K_{d:RimK.RimA}K_{d:{RimK}^{1}.cdG}=K_{d:RimK.cdG}K_{d:{RimK}^{2}.RimA}$ |
| **Five state RimK** | $RimK+RimA \rightleftarrows{RimK}^{1}$  $RimK+cdG \rightleftarrows{RimK}^{2}$  ${RimK}^{1}+cdG \rightleftarrows{RimK}^{3}$  ${RimK}^{2}+RimA \rightleftarrows{RimK}^{3}$  ${RimK}+RimB \rightleftarrows{RimK}^{4}$  $RimK+ATP \rightleftarrows{RimK.ATP} \to RimK+ADP+P_{i}$  ${RimK}^{1}+ATP \rightleftarrows{{RimK}^{1}.ATP} \to{RimK}^{1}+ADP+P_{i}$  ${RimK}^{2}+ATP \rightleftarrows{{RimK}^{2}.ATP} \to{RimK}^{2}+ADP+P_{i}$  ${RimK}^{3}+ATP \rightleftarrows{{RimK}^{3}.ATP} \to{RimK}^{3}+ADP+P_{i}$  ${RimK}^{4}+ATP \rightleftarrows{{RimK}^{4}.ATP} \to{RimK}^{4}+ADP+P_{i}$ | $RimK+{RimK}^{1}+ {RimK}^{2}+ {RimK}^{3}+{RimK}^{4}={RimK}^{Total}$  $K_{d:RimK.RimA}K_{d:{RimK}^{1}.cdG}=K_{d:RimK.cdG}K_{d:{RimK}^{2}.RimA}$ |
