# Supplementary material for: Control of mRNA translation by dynamic ribosome modification
Source: PLoS Genet. 2020 Jun 25;16(6):e1008837. doi: 10.1371/journal.pgen.1008837 (PMC7343187; doi:10.1371/journal.pgen.1008837)
Supplement: S6 Table — (DOCX) [file pgen.1008837.s014.docx]

**S6 Table – Strains and Plasmids**

| Strains | Description | Reference |
| --- | --- | --- |
| P. fluorescens |  |  |
| SBW25 | Environmental *P. fluorescens* isolate | [1] |
| SBW25 ∆rimB | SBW25 with *rimB* (*PFLU_0262*) deleted | [2] |
| SBW25 ∆rimK | SBW25 with *rimK* (*PFLU_0261*) deleted | [2] |
| SBW25 ∆rimBK | SBW25 with *rimBK* (*PFLU_0261-2*) deleted | This study |
| SBW25 rpsF_10glu_ | SBW25 with *rpsF_10glu_* allele | This study |
| SBW25 ∆rimB rpsF_10glu_ | SBW25 ∆*rimB* with *rpsF_10glu_* allele | This study |
| SBW25 ∆rimK rpsF_10glu_ | SBW25 ∆*rimK* with *rpsF_10glu_* allele | This study |
| SBW25 ∆rimBK rpsF_10glu_ | SBW25 ∆*rimBK* with *rpsF_10glu_* allele | This study |
| SBW25 ∆rimBK rpsF_4glu_ | SBW25 ∆*rimBK* with *rpsF_4glu_* allele | This study |
| SBW25 ∆hfq | SBW25 with *hfq* (*PFLU_0520*) deleted | [2] |
| SBW25 hfq-flag | SBW25 with *hfq* (*PFLU_0520*) C-terminally flag-tagged | This study |
| SBW25 ∆rimK hfq-flag | SBW25 ∆*rimK* with *hfq* C-terminally flag-tagged | This study |
| E. coli |  |  |
| BL21-(DE3) pLysS | Sm^R^, K12 *recF143 lacI^q^ lacZΔ.M15*, *xylA,* pLysS | Novagen |
| DH5α | *endA*1, *hsdR*17(r_K_-m_K_+), *supE*44, *recA*1, *gyrA* (Nal^r^), *relA*1, Δ(*lacIZYA-argF*) U169, *deoR*, Φ80*dlacΔ(lacZ)M15* | [3] |
| Plasmids |  |  |
| pSUB11 | Amplification vector for *flag-*FRT-Kan^R^-FRT cassette | [4] |
| pTS1 | Tet^R^, suicide vector; *ColE1*-replicon, *IncP-1, Mob, lacZ* | [5] |
| pTS1-rimBK | pTS1 with Δ*rimBK* construct as *Mfe*I-*Bam*HI fragment | This study |
| pTS1-rpsF_10glu_/_4glu_ | pTS1 with *rpsF_10glu_*/_4glu_ alleles as *Xho*I*-Bam*HI fragments | This study |
| pTS1-hfq_flag_ | pTS1 with *hfq_flag_* allele as *Nde*I*-Bam*HI fragment | This study |
| pETNdeM-11 | Km^R^, purification vector, N-terminal His_6_-tag | [6] |
| pETM11-rimA/B | pET*Nde*M-11 with SBW25 *rimA/B* as *Nde*I-*Xho*I fragments | (2) |
| pETM11-rpsF | pET*Nde*M-11 with *rpsF* allele as *Nde*I-*Xho*I fragment | [2] |
| pETM11-rpsF_10glu_ | pET*Nde*M-11 with *rpsF_10glu_* allele as *Nde*I-*Xho*I fragment | This study |
| pETM11-rimA_E47A_ | pET*Nde*M-11 with *rimA_E47A_* allele as *Nde*I-*Xho*I fragment | This study |
| pET42b(+) | Km^R^, purification vector, C-terminal His_6_-tag | Novagen |
| pET42b(+)-rimK | pET42b(+) with SBW25 *rimA* as *Nde*I-*Xho*I fragments | [2] |

**References**

1. Rainey PB, Bailey MJ. Physical and genetic map of the Pseudomonas fluorescens SBW25 chromosome. Mol Microbiol. 1996;19(3):521-33. Epub 1996/02/01. PubMed PMID: 8830243.

2. Little RH, Grenga L, Saalbach G, Howat AM, Pfeilmeier S, Trampari E, et al. Adaptive Remodeling of the Bacterial Proteome by Specific Ribosomal Modification Regulates Pseudomonas Infection and Niche Colonisation. PLoS Genet. 2016;12(2):e1005837. doi: 10.1371/journal.pgen.1005837. PubMed PMID: 26845436.

3. Woodcock DM, Crowther PJ, Doherty J, Jefferson S, DeCruz E, Noyer-Weidner M, et al. Quantitative evaluation of Escherichia coli host strains for tolerance to cytosine methylation in plasmid and phage recombinants. Nucleic Acids Res. 1989;17(9):3469-78. PubMed PMID: 2657660.

4. Yu D, Ellis HM, Lee EC, Jenkins NA, Copeland NG, Court DL. An efficient recombination system for chromosome engineering in Escherichia coli. Proc Natl Acad Sci U S A. 2000;97(11):5978-83. PubMed PMID: 10811905.

5. Scott TA, Heine D, Qin Z, Wilkinson B. An L-threonine transaldolase is required for L-threo-beta-hydroxy-alpha-amino acid assembly during obafluorin biosynthesis. Nature Communications. 2017;8. doi: ARTN 1593510.1038/ncomms15935. PubMed PMID: WOS:000404029700001.

6. Little R, Salinas P, Slavny P, Clarke TA, Dixon R. Substitutions in the redox-sensing PAS domain of the NifL regulatory protein define an inter-subunit pathway for redox signal transmission. Mol Microbiol. 2011;82(1):222-35. Epub 2011/08/23. doi: 10.1111/j.1365-2958.2011.07812.x. PubMed PMID: 21854469.
