# Supplementary material for: Control of mRNA translation by dynamic ribosome modification
Source: PLoS Genet. 2020 Jun 25;16(6):e1008837. doi: 10.1371/journal.pgen.1008837 (PMC7343187; doi:10.1371/journal.pgen.1008837)
Supplement: S7 Table — (DOCX) [file pgen.1008837.s015.docx]

| S7 Table Primers used in this study | | |
| --- | --- | --- |
| Number/Name | **Sequence (5’→3’)** | **Description** |
| 1/ 0263BacFor | GAGGATCCCGTGACCGACTTCCCCCTTTCAC | Forward probe for *PFLU0263* (*rimA*) |
| 2/ 0261BacRev | GTCGGTACCGCCCTTGCCTTTGGTCCGAG | Reverse probe for *PFLU0261* (*rimK*) |
| 3/ rimA-GSP1 | TCGAAAGGTGGGTTTGCATC | 5’ RACE gene-specific primer for cDNA synthesis |
| 4/ rimA-GSP2 | TTGCTTTCGTTGACCTGGT | 5’ RACE nested gene-specific primer for cDNA amplification |
| 5/ 0261RT-PCR1 | GCAAGCCGCTGGAAGGGTTTGATG | *PFLU0261* (*rimK*) forward primer for qPCR |
| 6/ 0261RT-PCR2 | GCAACTGCAGCGAACGCAACTTG | *PFLU0261* (*rimK*) reverse primer for qPCR |
| 7/ 0261NdeFor | CAGAAGCCATATGAAGATTGCTGTGCTGTCG | *PFLU0261* (rimK) purification, forward primer |
| 8/ 0261XhoRev | GGTACTCGAGGCCCTTGCCTTTGGTCCGAGTC | *PFLU0261* (*rimK*) purification, reverse primer |
| 9/ 0262NdeFor | CAGAAGCCATATGAAGACATTTGACCATTTG | *PFLU0262* (*rimB*) purification, forward primer |
| 10/ 0262XhoRev2 | GGTACTCGAGTCATGCAGCACCTGGGGC | *PFLU0262* (*rimB*) purification, reverse primer |
| 11/ 0263NdeFor | CAGAAGCCATATGACCGACTTCCCCCTTTCAC | *PFLU0263* (*rimA*) purification, forward primer |
| 12/ 0263XhoRev2 | GGTACTCGAGTCAATTCGCCCAGGCCTGGGG | *PFLU0263* (*rimA*) purification, reverse primer |
| 13/ ColiRpsFNdeFor | CAGAAGCCATATGCGTCATTACGAAATCGTT | *E coli* *rpsF* purification, forward primer |
| 14/ ColiRpsXhoRev2 | GGTACTCGAGTTACTCTTCAGAATCCCCAGC | *E. coli rpsF* purification, reverse primer |
| 15/ 0263E47AFor | CGCCCACGCAGCCTTGGTGCGC | PFLU0263 (RimA) forward mutagenic primer |
| 16/ 0263E47ARev | GCGCACCAAGGCTGCGTGGGCG | PFLU0263 (RimA) reverse mutagenic primer |
| 17/ 0533NdeFor | CAGAAGCCATATGCGTCATTACGAAATC | *PFLU0533* (*rpsF*) purification, forward primer |
| 18/ 0533XhoRev10Glu | GGTACTCGAGTTATTCTTCTTCTTCTTCTTCTTCTTCTTCTTCCTCGTCAGCGTTATCGC | *PFLU0533* (*rpsF*) purification with ‘10glu’ tail, reverse primer |
| 19/ PFLU0261UPF | CGGGATCCTATTGGTTCGCCAAGC | *PFLU0261* (*rimK*) deletion; forward primer, upstream |
| 20/ PFLU0261UPR | CGTCTAGACAGACGCGGGTTTCGCGAC | *PFLU0261* (*rimK*) deletion; reverse primer, upstream |
| 21/ PFLU0261DNF | CGTCTAGATGATTAAACCCTGCTTTTG | *PFLU0261* (*rimK*) deletion; forward primer, downstream |
| 22/ PFLU0261DNR | CGGAATTCGACGGACTTGGTGCGTG | *PFLU0261* (*rimK*) deletion; reverse primer, downstream |
| 23/ PFLU0262UPR | CGTCTAGACAAATGGTCAAATGTC | *PFLU0261* (*rimB*) deletion; reverse primer, upstream |
| 24/ PFLU0262DNF | CGTCTAGAGCCCCAGGTGCTGCATG | *PFLU0262* (*rimB*) deletion; forward primer, downstream |
| 25/ PFLU0262DNR | CGGAATTCATTGCCGCAATCACCTTG | *PFLU0262* (*rimB*) deletion; reverse primer, downstream |
| 26/ hfq1NdeIF | GGAATTCCATATGATGTCAAAAGGGCATTCGC | *PFLU0520* (*hfQ*) amplification. Forward primer |
| 27/ hfq258SalIR | GCGTCGACGGCGTTACCTGGCTCAGCG | *PFLU0520* (*hfQ*) amplification. Reverse primer |
| 28/ 3xFLAGSalIF | GCGTCGACGACTACAAGGACCACGAC | Amplification of the FLAG epitope sequence. Forward primer. |
| 29/ 3xFLAGXbaIR | GCTCTAGATCACTTGTCGTCGTCGTCC | Amplification of the FLAG epitope sequence. Reverse primer. |
| 30/ Inter+hflXXbaIF | GCTCTAGATAGGAGTCTCCTTTGTTCT | Amplification from STOP codon of *hfQ* into *hflX*. Forward primer. |
| 31/ hflX258BamHIR | CGGGATCCGATTGAAGATCACCAGATC | Partial amplification of *hflX*. Reverse primer |
| 32/ 25RpsFupF | CCGCTCGAGGAGCATTCATGCGTCATTAC | *PFLU0533* (*rpsF*) upstream forward primer |
| 33/ 25RpsFgluF | GAGGAGGAGGAGTAATAATCCACGGACCTTTTAAG | Addition of four glutamates to *PFLU0533* (*rpsF*). Forward primer |
| 34/ 25RpsFgluR | TTACTCCTCCTCCTCCTCGTCAGCGTTATCGCTG | Addition of four glutamates to *PFLU0533* (*rpsF*). Reverse primer |
| 35/ 25RpsDnR | CGGGATCCCCAATACAACAACGGC | *PFLU0533* (*rpsF*) upstream downstream primer |
| 36/ 10gluRpsFfor | GAGGAGGAGGAGGAGGAGGAGGAGGAGGAGTAATCCACGGACCTTTTAAG | Addition of ten glutamates to *PFLU0533* (*rpsF*). Forward primer |
| 37/ 25RpsFgluF2 | TTACTCCTCCTCCTCCTCCTCCTCCTCCTCCTCCTCGTCAGCGTTATCGCTG | Addition of ten glutamates to *PFLU0533* (*rpsF*). Reverse primer |
