# Supplementary material for: Control of mRNA translation by dynamic ribosome modification
Source: PLoS Genet. 2020 Jun 25;16(6):e1008837. doi: 10.1371/journal.pgen.1008837 (PMC7343187; doi:10.1371/journal.pgen.1008837)
Supplement: Attachment — Submitted filename: 103790_1_rebuttal_1720114_q8db0v-2.pdf [file pgen.1008837.s016.pdf]

Reviewer #1 (Evidence, reproducibility and clarity (Required)):
General comments
The manuscript 'Second messenger control of mRNA translation by dynamic ribosome modification' is a very interesting follow up on the research performed by the authors published in 2016. Here, the authors continue their study by determining the impact of the intricate RimABK pathway in Pseudomonas fluorescens on translational reprogramming by controlled modification of ribosomal protein S6 in response to environmental signals. The manuscript is interesting and well written, and the results are sound. However, in my opinion the general conclusion is not supported by experimental data and leaves several potential explanations open. Thus, I suggest to either perform in vitro translation experiments using ribosomes equipped with glutamated S6 to validate translational selectivity, or to soften the language on the working model shown in Figure 12.

The authors would like to thank reviewer 1 for their detailed review of our manuscript. We agree with the reviewer that alternative explanations are possible for the translational changes linked specifically to glutamation, as opposed to *rimBK* deletion. Our intention when writing the discussion was to clearly distinguish glutamation-specific effects from the large number of indirect translational changes associated with Hfq disruption and other uncharacterised consequences of *rimBK* deletion. With hindsight, we acknowledge that the discussion and the model in figure 12 should more clearly outline the possible alternative causes for the observed glutamation-specific translational regulation. We have modified the discussion and figure 12 (now figure 10) accordingly.

The reviewer further suggests that we perform in vitro translation experiments using ribosomes equipped with glutamated S6, to prove that glutamation controls translation directly. This is an excellent suggestion that would clarify this important point, and we will certainly attempt it as part of our future analysis of the Rim system. However, at this stage we feel these experiments are beyond the intended scope of this paper, which is to describe the signal inputs and mechanism of the RimABK system and to show evidence for both specific and secondary translational effects of ribosome modification.

Specific comments
Figure 1 and S1: The RT-PCR analysis shown here does not allow excluding transcription initiation at alternative promoters downstream of the one determined by 5'-RACE. However, an alternative promoter might contribute to relative ratios between the rimA, rimB, and rimK mRNAs. A Northern blot and/or primer extension analysis could clarify this assumption and would give more detailed insights into the specific regulation.

The reviewer is correct that alternative *rim* promoters could exist downstream of the amplified 5'-RACE region. To test this hypothesis, we conducted additional RT-PCR experiments to measure expression of *rimA* (the third gene of the polycistronic *rimABK* operon) in the same set of conditions as tested for *rimK*. Relative levels of *rimA* mRNA do not substantially differ from those seen for *rimK*, strongly suggesting that the promoter upstream of *rimK* controls expression of all three *rim* genes. We have added this dataset to figure S1 and have modified the relevant sections of the text.

Figure 2B: I'm confused by the results shown here! I do only see a reduction of RpsF in the presence of RimA, RimK and cdG. What indicates the modification? Please, explain the interpretation of the result in more detail. Shouldn't the modified RpsF shift due to the addition of glutamate residues?

The uncontrolled activity of RimK acting in the absence of RimB (e.g. the experiment represented in Fig 2B) typically results in a reduction of the unmodified RpsF fraction in the reaction, replaced with RpsF proteins with widely varying numbers of glutamate residues attached to their C-termini. The resulting modified RpsF fraction can appear as a smear of protein density throughout the gel. We have clarified the text surrounding figure 2 to make this more explicit.

Figure 2C: Why does the RpsF modification lead to a supershift? How many glutamate residues are added? Is the smear visible in lane 4 (RpsF+RimK) representing already the slightly modified RpsF protein, which upon

addition of RimA results in a supershift? For all SDS-Page analyses shown in the manuscript the validation of the glutamation using the antibodies specific against poly-glutamate would be a great asset to facilitate their interpretation.

Pseudomonas fluorescens RimK appears to have unregulated ligase activity, with many hundreds of glutamates being added to each RpsF protein in the absence of RimB cleavage. In our 2016 paper (Little et al., PLoS Genetics) we use radiolabelled glutamate incorporation and mass spectrometry to show that the supershifted protein smear is composed entirely of RpsF units with C-terminal glutamate tails of varying length. (It is interesting to note that *E. coli* RimK, which does not have an accompanying RimB protease, can only add 4-15 glutamates to each RpsF protein). We have modified the text slightly to make this clearer.

The reviewer's suggestion to stain the supershifted RpsF with the poly-E antibody is interesting but would likely only reiterate our published results with radiolabelled glutamate (Little et al. 2016).

Lines 236-238: '...strongly suggesting that the proteomic changes we observe are an active response to modification of ribosomally-associated RpsF proteins.' This is an important suggestion as it allows a flexible and very fast integration of the external signals into a specialized protein synthesis. Thus, it definitely deserves further analysis! Considering that the purified RimA and RimK proteins are available, in vitro modification of RpsF in the context of the purified ribosome would be an important experiment and would greatly increase the quality of the paper. Up to now the selective or specialized translation is pure hypothesis and might also be explained by indirect effects via e.g. increased interaction between the ribosome and HFQ that might mediate interaction with certain mRNAs and thus stimulate their translation.

We agree with the reviewer that direct measurement of translational changes *in vitro* would tell us a great deal about the mechanism of RimK regulation. This would enable us to confirm whether the glutamation-specific effect is direct, or if it functions through an as-yet uncharacterised indirect mechanism (such as interaction with another translational regulator). As stated above we feel these major experiments are beyond the scope of the current manuscript, although we are keen to do them (as part of a planned structural biology investigation of modified ribosomes). As stated elsewhere in our response, we have extensively revised the discussion text and figure 12 to clarify the limits of our current understanding and highlight the different potential regulatory routes for RpsF glutamation.

Lines 322: '...into a single output: the proportion of all ribosomally-associated RpsF proteins that have C-terminal poly-glutamate tails.' Considering the identification of a group of genes whose translation is altered by rimBK deletion, but not by RpsF glutamation (Class 1, Fig 11B), I would suggest softening this statement. If I interpret the data correctly, they pinpoint to a moonlighting function of the rim-pathway that does not target RpsF!

The genes whose translation is affected by *rimBK* deletion, but not by RpsF glutamation specifically, include all those genes whose translation is indirectly affected by downstream translational regulators, or through interaction with another affected gene target. As expected, there is substantial overlap between the *rimBK* and *hfq* translatomes (Grenga et al. 2017): this analysis can be included in the manuscript as a supplementary table if requested. Importantly, there is very little overlap between the Hfq translatome and those genes that are affected specifically by RpsF glutamation. One possibility is that Hfq interacts with RimK at the ribosome, and the loss of the RimK protein is a major factor in destabilising Hfq function in the *ΔrimK* mutant. We have modified figure 12 (now figure 10) and expanded the discussion to include this hypothesis.

While we cannot exclude the possibility that RimK has other cellular targets in SBW25, we think this is unlikely to be a major cause for the results we see here. We have carefully examined the C-terminal peptides of proteins detected in our various proteomic assays and are confident that RpsF is the sole target of RimK in SBW25 under the conditions we tested. We also directly tested RimK interaction with purified Hfq and confirmed that Hfq is not a direct target of RimK modification.

Lines 377-76: '...distinguishing features in the primary or predicted secondary structures of the Rim-mRNAs...' As mentioned already above several indirect options are still open that could confer selectivity to the ribosome.

As stated above, the discussion has been rewritten to more completely reference the possible mechanisms by which RpsF glutamation may lead to translational regulation.

Reviewer #1 (Significance (Required)):
The key concept of the manuscript namely the impact of the intricate RimABK pathway in Pseudomonas fluorescens on translational reprogramming by controlled modification of ribosomal protein S6 in response to environmental signals is novel and will significantly impact the field.

Reviewer #2 (Evidence, reproducibility and clarity (Required)):
Summary
The main question addressed by this research is how bacteria adapt to rhizospheric niche through the RimK ATPase glutaminase. This enzyme post-transcriptionally modifies the ribosomal protein RpsF in a process of complex regulation. Regulation is mediated by c-di-GMP that is degraded by the phosphodiesterase RimA and the protease RimB exerts a role opposite of RimK. Novel findings include the finding of RimK acting as a four-state ATPase, depending on the binding of RimA, c-di-GMP or both. Another important finding is the opposite roles of RimK and RimB on the glutamation/deglutamation of RpsF and the tendency to a steady state of four glutamate residues in the RpsF protein. The authors also use proteomics to determine the effect of glutamation, specially at low temperature and under nutrient limitation.

We thank the reviewer for their positive review of the manuscript and address their comments below.

Major comment
In my opinion, the results obtained with the Hfq regulation by RimK blur the message. I firmly think that the Ms is very solid with the results obtained in relation with the RimABK/RpsF regulation in P. fluorescens shown as a model in the Figure 12. Moreover, in this final model presented by the authors (fig. 12) they not included the results related with Hfq. These results could be part of another paper.

We agree with the reviewer that the Hfq independent effects of RpsF are an exciting finding and should be a major focus of the paper. That said, we feel that the additional work we have done showing how Hfq is affected by RimK should also be retained in the manuscript in some form. Our data (e.g. figure 8) indicate that Hfq is responsible for a large (indirect) fraction of the *ΔrimK* phenotype, so understanding how it is affected is important to understand how RimK functions. Based on comments from reviewers 2 and 3 we have reviewed the manuscript text (including data on Hfq) to make the narrative as focussed and clear as possible. We have also redesigned figure 12 (now Fig 10) to consider comments from all three reviewers and have changed the text in the discussion to match this.

Minor comments
In figure 4A, what is lane 5?

Lane 5 contains RimB without ADP. The figure legend has been modified accordingly, and we thank the reviewer for highlighting this error.

Line 159 change "suppression of RimK band-shifting" by "suppression of RpsF band shifting"

This has been fixed.

Reviewer #2 (Significance (Required)):
The Ms. is very interesting and deeply describes the relation between environmental conditions, c-di GMP second messenger and the RpsF ribosomal protein posttranscriptional modification in order to respond to low temperatures and changes in nutrient availability. The research developed in this manuscript is original and novel in the field and includes new advances in the signal transduction pathways implicated in the regulation of bacteria adaption to the environment. Besides, the research design and technical methodology is original and includes multidisciplinary approaches of interest to the research community in general.

Reviewer #3 (Evidence, reproducibility and clarity (Required)):
Summary
Post-transcriptional control of protein abundance is an important yet poorly examined regulatory process by which bacteria respond to signals found in the environments they grow in. The authors' team have previously identified, described and published details around a novel regulatory pathway involving the ribosomal modification protein RimK, regulator proteins RimA and RimB, and the widespread bacterial second messenger cyclic-di-GMP (cdG). In the current manuscript builds considerably on those previous findings and goes several steps further, through various approaches including protein biochemistry, computational modelling, quantitative proteomics and ribosomal profiling, the authors described how the RimABK pathway as a novel translator system that controls bacterial adaption to the rhizosphere in the bacterium Pseudomonas fluorescens. They show that the system achieves this through specific controlled modification of the ribosomal protein RpsF. I read the article with excitement and overall the manuscript describes an extensive data set that will be of considerable interest to many readers in several fields. However, I have made a few points below that the authors need to take on board and address. If these issues are addressed, I believe it will make the presented data much clearer to the reader, tidy up a few ambiguities and make the article a little more accessible to many non-specialist readers.

We thank the reviewer for their thorough and positive assessment of the manuscript. We address their specific points below.

Major Comments
1) The major finding described in the manuscript and the one that will be of significant interest to reader is that a novel post-translational ribosomal modification regulatory mechanism involving Rim system controls bacterial adaption. The second messenger cdG only plays a small part in this complicated process. Therefore, I believe the title needs to be revised to capture the scope and key findings of the manuscript.

We are happy to change the title along the lines suggested by the reviewer. We propose: *Control of mRNA translation by dynamic ribosome modification* as a new title.

2) The authors present a lot of interesting data; however, I found the manuscript a bit of a dense read. I find the key findings are diluted within the text. I would ask that the authors to make it a little more focused. For example, on the regulatory role of RimK and its influence on Hfq and RpsF has been detailed previously so could be placed in supporting information and briefly mention when required. Also, the experiments on the pvdIJ pathway could be removed or placed in supporting information as they are not the main focus of the manuscript. Fig 5 and 6 could be combined as one figure as well.

We have modified the manuscript throughout to make it clearer, more concise, and to focus as much as possible on new findings rather than reiterating what we showed in our last manuscript. In line with the reviewers' recommendation, we have moved the *pvdIJ* data into the supplementary material (Fig S3) and merged figures 5 and 6 into one. In addition, to support our data on the importance of RpsF glutamation for ribosomal regulation we used Western blotting to confirm that $RpsF_{4/10glu}$ variants incorporate normally into SBW25 ribosomes *in vivo* (added as supplemental data Fig S5).

As stated elsewhere, we feel that key data on the relationship between Hfq and RimK should remain in the main manuscript, although we have reviewed the text thoroughly to try to ensure it is as focussed as possible and have moved some results to supplementary material as suggested.

3) The authors propose a four-state kinetic model for RimK ATPase activity with RimA and cdG (described in Fig2 and Table S1). However, later in the manuscript the authors demonstrate that RimB also stimulates RimK ATPase activity, but this seems to have smaller impact than RimA and cdG (Fig 2E, Fig 3A). Why RimB was not included in the ATPase kinetic model of RimK? Does including the RimB data suggest there might be more conformational states for RimK?

Thank you for raising this point. The reviewer is correct in that this data does indeed suggest another level of ATPase activity of RimK. We have added text to the manuscript to reflect this. We have also extended the supplemental Table S1 to include these equations.

4) The authors claim that the suppressive effect of cdG on RimK was depended on the enzyme activity (PDE domain) of RimA. This was tested using an enzymatically inactive RimA variant (RimA-E47A). However, in Fig 3E the amount of RimA-E47A used in the assay seems to be significantly less than wildtype RimA. Additionally, in Fig 2B, the authors show that addition of cdG also stimulates RpsF modification with or without RimA (lane 4-6). I would ask the authors to clarify these points.

It is difficult to directly compare protein variants due to differences in solubility post-purification. Due to difficulties in purifying this (less soluble) form of RimA, co-purifying contaminants have also probably influenced the determination of RimA-E47A concentration to some extent. This restricts us to making largely qualitative statements about protein function, as we do here. Despite its poor solubility and low concentration, RimA-E47A is still able to stimulate RimK. Furthermore, the relatively low concentration of RimA-E47A in our assays would render it at least as susceptible to any effects of cdG addition as WT RimA, meaning we can be confident that cdG has no effect on RimK stimulation by this variant.

Our model incorporates direct stimulation of RimK by cdG alongside its effect on RimA. We show evidence for this in this manuscript and in our 2016 paper.

5) The authors claim that high levels of cdG increase the ratio of RimB protease activity to RimK glutamate ligase activity. However, there is no experiment to provide direct evidence to support this. Please tone down the language used or provide evidence. On the same point Fig 6 was not explained in the main text to support this conclusion. Please include an explanation.

The hypothesis that high cdG levels favour RimB activity over RimK stems from the observation that cdG suppresses RimK activity (by abolishing RimA stimulation) but does not affect RimB. We have data showing that increasing cdG levels suppresses RpsF band shifting *in vitro* in an assay containing all three Rim proteins (Fig 4). However, we agree the hypothesis that cdG controls the ratio of RimB to RimK activity by controlling the activity of RimK currently lacks explicit, direct evidence and we have modified the text to tone down the language.

An explanation for Figure 6 (now 5b) has been added to the manuscript as requested.

6) In some of the figures/images, for example, Fig2B and Fig 3E, RimA is shown as a major band. However, in other figures/images, for example, Fig 2D, Fig 3D, RimA seems to be two bands. The authors should explain the reason for this.

Based on extensive experimentation, we are confident that the second band present in some of our assays is a cleavage product of RimA. This is an experimental artefact that is linked to concentration and protein stability *in vitro*. We must stress that the presence of an inactive fraction of RimA in our assays does not affect the conclusions we are able to draw from these experiments. A note has been added to the relevant section of the text.

Minor comments

1) Line 151, should be RpsF band-shifting instead of RimK.
2) Fig 4A there is no legend for lane 5, which made it very difficult to understand the data presented.

Please see above. These two minor errors will be fixed.

3) The layout of some figures could be improved.

We have revised the layout of several figures, in line with the reviewer's suggestion.

4) If it is possible to have Fig 11 as a Venn diagram or some intuitive diagram, it will help the readers gain access to the data and understand the results.

We respectfully disagree with the reviewer here. We have tried several different presentation styles for these data, but ultimately considered scatter charts to be the most effective, in line with our previous study of Hfq regulation in *Pseudomonas* (Grenga et al. Frontiers in Microbiology 2017).

5) Fig 12 is very neatly laid out. However, I don't feel it captures the dynamic nature of the system. I am just wondering if the authors could break it down so that it describes the changes relating to environmental conditions and/or different cdG levels?

Figure 12 (now Figure 10) has been modified to reflect to comments of all three reviewers.

Reviewer #3 (Significance (Required)):
The manuscript provides detailed evidence to demonstrate a dynamic, post-translational ribosomal modification mechanism which is an important feature of prokaryotic (potentially archaeal and eukaryotic) environmental adaptation. This is an exciting manuscript and one many will wish to read. The data provided will be of interest to scientists working in many fields including microbiology, biochemistry and plant pathology.

I have several areas of expertise including genomics, molecular microbiology, small molecule signalling and regulation, micro-host interaction, adaptation, virulence and pathogenies.
